# Quantitative Analysis of Methylated Adenosine Modifications Revealed Increased Levels of *N*^6^-Methyladenosine (m^6^A) and *N*^6^,2′-*O*-Dimethyladenosine (m^6^Am) in Serum From Colorectal Cancer and Gastric Cancer Patients

**DOI:** 10.3389/fcell.2021.694673

**Published:** 2021-07-26

**Authors:** Yiqiu Hu, Zhihao Fang, Jiayi Mu, Yanqin Huang, Shu Zheng, Ying Yuan, Cheng Guo

**Affiliations:** ^1^Cancer Institute (Key Laboratory of Cancer Prevention and Intervention, China National Ministry of Education), The Second Affiliated Hospital, Zhejiang University School of Medicine, Hangzhou, China; ^2^Department of Medical Oncology, The Second Affiliated Hospital, Zhejiang University School of Medicine, Hangzhou, China; ^3^Cancer Center, Zhejiang University, Hangzhou, China

**Keywords:** colorectal cancer, gastric cancer, RNA methylation, methylated adenosine, hydrophilic interaction liquid chromatography-tandem mass spectrometry, biomarker

## Abstract

Colorectal cancer and gastric cancer are the most prevalent gastrointestinal malignancies worldwide, and early detection of these cancers is crucial to reduce their incidence and mortality. RNA methylation plays an important regulatory role in a variety of physiological activities, and it has drawn great attention in recent years. Methylated adenosine (A) modifications such as *N*^6^-methyladenosine (m^6^A), *N*^1^-methyladenosine (m^1^A), 2′-*O*-methyladenosine (Am), *N*^6^,2′-*O*-dimethyladenosine (m^6^Am), and *N*^6^,*N*^6^-dimethyladenosine (m^6^_2_A) are typical epigenetic markers of RNA, and they are closely correlated to various diseases including cancer. Serum is a valuable source of biofluid for biomarker discovery, and determination of these adenosine modifications in human serum is desirable since they are emerging biomarkers for detection of diseases. In this work, a targeted quantitative analysis method using hydrophilic interaction liquid chromatography–tandem mass spectrometry (HILIC-MS/MS) was developed and utilized to analyze these methylated adenosine modifications in serum samples. The concentration differences between the healthy volunteers and cancer patients were evaluated by Mann–Whitney test, and receiver operator characteristic (ROC) curve analysis was performed to access the potential of these nucleosides as biomarkers. We demonstrated the presence of the m^6^Am in human serum for the first time, and we successfully quantified the concentrations of A, m^6^A, m^1^A, and m^6^Am in serum samples from 99 healthy controls, 51 colorectal cancer patients, and 27 gastric cancer patients. We found that the levels of m^6^A and m^6^Am in serum were both increased in colorectal cancer or gastric cancer patients, compared to that in healthy controls. These results indicate that m^6^A and m^6^Am in serum may act as potential biomarkers for early detection and prognosis of colorectal cancer and gastric cancer. In addition, the present work will stimulate investigations on the effects of adenosine methylation on the initiation and progression of colorectal cancer and gastric cancer.

## Introduction

Colorectal cancer and gastric cancer are two common malignancies that are the second and third causes of cancer-related deaths all over the world, respectively (Brenner et al., 2014; Siegel et al., 2017; Bray et al., 2018). The incidence and mortality of these two types of cancer have been rising due to the difficulties in early detection. Detection of these cancers in the early stage has the advantage of improving survival rate and reducing costs of patients. Currently, the screening and diagnosis of colorectal cancer and gastric cancer mainly depend on results of colonoscopy and gastroscopy, respectively, which are both invasive to patients, leading to low compliance. From this point of view, it is essential to discover novel non-invasive biomarkers for early detection of colorectal cancer and gastric cancer to elongate the survival time and decrease the pains of patients.

Posttranscriptional modifications of RNA play crucial regulatory roles in a number of physiological activities (Feinberg, 2018; Bates, 2020). Until now, more than 170 chemical modifications of RNA have been identified, and these modifications are involved in the regulation of RNA functions (Jonkhout et al., 2017; Ontiveros et al., 2019). Among these chemical modifications, RNA methylation modifications have attracted great attention since accumulating evidences have been obtained to confirm RNA methylation as a novel layer of epigenetic alteration. Although methylation modifications in RNA had been identified in the 1970s (Dubin and Taylor, 1975; Perry et al., 1975), understanding of the functions of RNA methylation was limited until the identification of regulatory proteins such as fat mass and obesity-associated protein (FTO) (Jia et al., 2011). Through their unique regulatory proteins including “writers,” “erasers,” and “readers,” RNA methylation modifications display distinct features associated with many key cellular functions such as splicing, stability, and translation of RNA (Boo and Kim, 2020; Shi et al., 2020). And it has been revealed that RNA methylation participates in the initiation and progression of a number of diseases including cancer (Fang et al., 2021).

As a typical product of RNA methylation, *N*^6^-methyladenosine (m^6^A) is the most predominant modification in mRNA (Chen et al., 2019). The level of m^6^A is dynamic and reversible, and this is ascribed to the regulation of m^6^A methyltransferases and demethylases (Dominissini et al., 2012; Meyer and Jaffrey, 2017). The aberrant level of m^6^A modification is closely related to tumorigenesis and development (Lan et al., 2019; Li et al., 2019; Zhang et al., 2019; Liu et al., 2020; Wang et al., 2020a, b; Jiang et al., 2021). In recent years, other methylated adenosine modifications such as *N*^1^-methyladenosine (m^1^A), 2′-*O*-methyladenosine (Am), *N*^6^,2′-*O*-dimethyladenosine (m^6^Am), and *N*^6^,*N*^6^-dimethyladenosine (m^6^_2_A) have also been revealed to play critical roles in the pathogenesis of various cancers (Safra et al., 2017; Sendinc et al., 2019; Chen et al., 2020a; Dong and Cui, 2020; Alriquet et al., 2021). Therefore, these methylated adenosine modifications have great potential to be indicators for early detection of diseases.

Serum contains a large number of biomolecules, and it is a preferred body fluid in the realm of biomarker discovery (Djukovic et al., 2010; Chen et al., 2011; Huang et al., 2018; Ottosson et al., 2019; Stepien et al., 2021). In the past decades, multiple analytical approaches have been utilized for the analysis of modified nucleosides (Beale et al., 2018; Farrokhi et al., 2018; Pero-Gascon et al., 2018; Guo et al., 2019). Compared with other analytical techniques, liquid chromatography tandem mass spectrometry (LC–MS/MS) is more favored to be used for biomarker discovery due to its great advantages in selectivity, sensitivity, accuracy, and high throughput. The analysis time could be largely shortened when ultra-performance liquid chromatography (UPLC) was used, and thus, UPLC–MS/MS becomes the preferred method for the determination of large-scale clinical samples (Guo et al., 2016, 2018a,b, 2020; Chen et al., 2020b; Zheng et al., 2020; Su et al., 2021). In addition, hydrophilic interaction liquid chromatography (HILIC) has the advantages of higher sensitivity because the organic solvent-rich mobile phase is more volatile and can enhance desolvation and ionization efficiency in the ion source. In the present study, a fast, sensitive, simple, and reliable HILIC–MS/MS method for targeted quantitative detection of methylated adenosine modifications including A, m^6^A, m^1^A, Am, m^6^Am, and m^6^_2_A in human serum was established. By using the developed method, we revealed the presence of m^6^Am in human serum for the first time and quantified A, m^6^A, m^1^A, and m^6^Am in serum from 51 colorectal cancer patients, 27 gastric cancer patients, and 99 healthy volunteers. In addition, we carried out statistical analysis to compare the differences of these nucleosides between cancer patients and healthy controls and evaluated the potential of these adenosine modifications as biomarkers for early detection of colorectal cancer and gastric cancer.

## Materials and Methods

### Chemicals and Reagents

Chromatographic-grade methanol was obtained from J.T. Baker (Radnor, PA, United States). Acetonitrile of HPLC grade was bought from Merck KGaA (Darmstadt, Germany). A, m^6^A, m^1^A, Am, m^6^Am, m^6^_2_A, ^13^C_5_-adenosine ([^13^C_5_]A), D_3_-*N*^6^-methyladenosine ([D_3_]m^6^A), D_3_-*N*^1^-methyladenosine ([D_3_]m^1^A), and D_3_-*N*^6^-2′-O-dimethyladenosine ([D_3_]m^6^Am were gained from Toronto Research Chemical (Toronto, ON, Canada). Acetic acid (CH_3_COOH) was purchased from Fluka (Muskegon, MI, United States). Ammonium acetate and malic acid were bought from Sigma Aldrich (St Louis, MO, United States). Water was purified by using a Milli-Q water purification apparatus (Millipore, Milford, MA, United States).

### Instrumentation

Targeted quantitative analyses of adenosine and methylated adenosine were carried out on an Acquity UPLC system (Waters, Milford, MA, United States) coupled with a 4000 QTRAP mass spectrometer (AB SCIEX, Foster City, CA, United States). A Waters BEH HILIC column (2.1 × 100 mm, 1.7 μm) was implemented for chromatographic separation at room temperature. Mass spectrometer was operated in electrospray ionization (ESI) positive ion mode, and data were acquired by using multiple-reaction monitoring (MRM) mode. Data acquisition and processing were controlled by Analyst 1.6.3 software.

### Sample Collection

This research was approved by the Ethics Committee of the Second Affiliated Hospital, Zhejiang University School of Medicine (SAHZU). A total of 99 healthy controls (mean age of 50.3 ± 7.8 years, range 33–73 years) without serious infections or cancers, 51 patients with colorectal cancer (mean age of 63.2 ± 10.6 years, range 28–79 years), and 27 patients with gastric cancer (mean age of 65.9 ± 8.5 years, range 48–80 years) were recruited. The information of volunteers is shown in Table 1. All the subjects with cancers were confirmed by pathologist and did not undergo any type of therapy. Before participation, informed consent was provided by all volunteers. Then, the serum samples were collected in the early morning and reserved at −80°C until analysis.

**TABLE 1:** The basic information of individuals recruited in this study.

Group	Normal	Colorectal cancer	Gastric cancer
Number of cases	99	51	27
Gender (man/woman)	70/29	28/23	17/10
Age (year)	50.26 ± 7.82	63.16 ± 10.64	65.39 ± 8.53

### Sample Preparation

Serum samples of 100 μl were placed into a 1.5-ml centrifuge tube and spiked with 10 μl of isotope-labeled internal standards (IS) after being thawed in ice. In order to perfectly remove the protein, 330 μl of prechilled methanol/acetonitrile (2:1, v/v) was added, and the mixture was vortexed for 1 min. After being placed at −20°C for 2 h, the obtained mixture was centrifuged at 13,000 rpm at 4°C for 15 min. Subsequently, 352 μl of supernatant was evaporated to dryness under vacuum. Then, the dried samples were redissolved in 80 μl of acetonitrile/water (9:1, v/v). After vortexing for 10 s, ultrasonication for 15 s, and centrifuging at 13,000 rpm for 15 min at 4°C, in that order, 70 μl of the supernatant fraction was moved into the vial for HILIC–MS/MS detection.

### HILIC-MS/MS Analysis

The mobile phases were (A) H_2_O containing 10 mM ammonium acetate and 0.2% acetic acid and (B) acetonitrile containing 2 mM ammonium acetate, 0.2% acetic acid, and 0.05 mM malic acid. The desired sample separation was achieved by the optimized LC gradient program as follows: 0 min, 95% B; 3 min, 94% B; 3.5 min, 60% B; 5.5 min, 60% B; 6 min, 94% B; and 12.5 min, 94% B. The injection volume was 5 μl, and each sample was measured twice. The samples were maintained at 4°C. To minimize interference of the mass spectrometer, a switching valve was used, and the eluents from the column during 1.5–3.8 and 4.8–5.6 min were introduced into the ion source. The ion spray voltage was optimized as 5.5 kV. The ion source temperature (TEM) was set at 550°C. The pressure of ion source gas 1 (GS1) and ion source gas 2 (GS2) were both set at 50 psi. The curtain gas (CUR) was set at 40 psi. The dwell time was 45 ms for each ion transition.

For targeted quantitative analysis, the transitions of *m*/*z* 268.1→136.0 (A), *m*/*z* 282.1→150.0 (m^6^A and m^1^A), *m*/*z* 282.1→136.0 (Am), *m*/*z* 296.1→150.0 (m^6^Am), and *m*/*z* 296.1→164.0 (m^6^_2_A) were monitored. For isotope-labeled internal standards, the transitions of *m*/*z* 273.1→136.0 ([^13^C_5_]A), *m*/*z* 285.1→153.0 ([D_3_]m^6^A and [D_3_]m^1^A), and *m*/*z* 299.1→153.0 ([D_3_]m^6^Am) were monitored. The optimized MRM parameters for these nucleosides including declustering potential (DP), entrance potential (EP), collision energy (CE), and collision cell exit potential (CXP) are listed in Supplementary Table 1.

### Method Validation

In order to establish the calibration curves, different concentrations of the working solutions of nucleoside standards mixed with internal standards (IS) were made. The final concentrations of IS were as follows: [^13^C_5_]A 25 nM, [D_3_]m^6^A 2.5 nM, [D_3_]m^1^A 125 nM, and [D_3_]m^6^Am 1.25 nM. By measuring the peak area ratio of the nucleosides to the corresponding IS (y), against the concentration of the analyte (x), the linearities were constructed as *y* = ax + b. In addition, the limit of detection (LOD) and limit of quantification (LOQ) were obtained by analyzing the concentrations of the standard solutions with signal-to-noise ratios equal to 3 and 10, respectively.

For the purpose of evaluating extraction recovery, serum samples were spiked with standards at three different levels of A (1, 5, and 50 nM), m^6^A (1, 5, and 50 nM), m^1^A (10, 100, and 500 nM), and m^6^Am (0.1, 1, and 5 nM). After addition of the IS solution, the serum samples were processed and measured as mentioned above. The recovery (R) was estimated by using the following formula: recovery = (concentration in spiked sample − concentration in original sample)/spiked concentration × 100%.

In order to evaluate intra-day and inter-day precision, the quality control (QC) samples at three different levels of A (1, 10, and 75 nM), m^6^A (1, 10, and 75 nM), m^1^A (10, 250, and 750 nM), and m^6^Am (0.1, 2.5, and 20 nM) were measured on the same day and three consecutive days, respectively. The accuracy was acquired by comparing the obtained concentration to the theoretical value.

In consideration of matrix effect evaluation, a slope comparison method was applied. By adding standard solutions and IS to the serum and pure solvent, respectively, different calibration curves were subsequently established, and their slopes were compared. The slope ratio of the calibration curve established in serum to that in pure solvent was described as the matrix effect.

### Statistics Analysis

Statistical analyses were performed by utilizing the SPSS statistics 24.0 software (IBM, Armonk, NY, United States). The concentration differences between healthy volunteers and cancer patients were assessed by the Mann–Whitney test, and a *p*-value less than 0.05 was significant. Receiver operating characteristic (ROC) analysis was used to assess the ability of these nucleosides to discriminate cancer patients from healthy controls.

## Results and Discussion

### Optimization of Chromatographic Conditions and Mass Spectrometry Parameters

**FIGURE 1:**
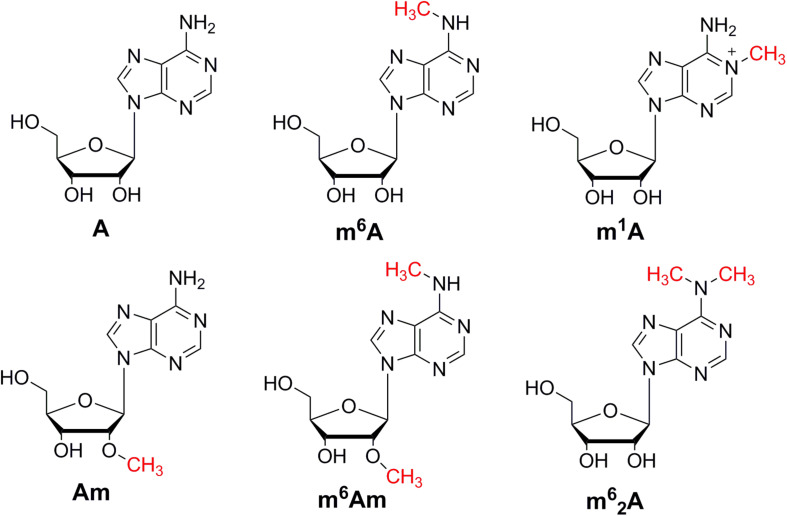
The chemical structures of A, m^6^A, m^1^A, m^6^Am, Am, and m^6^_2_A.

**FIGURE 2:**
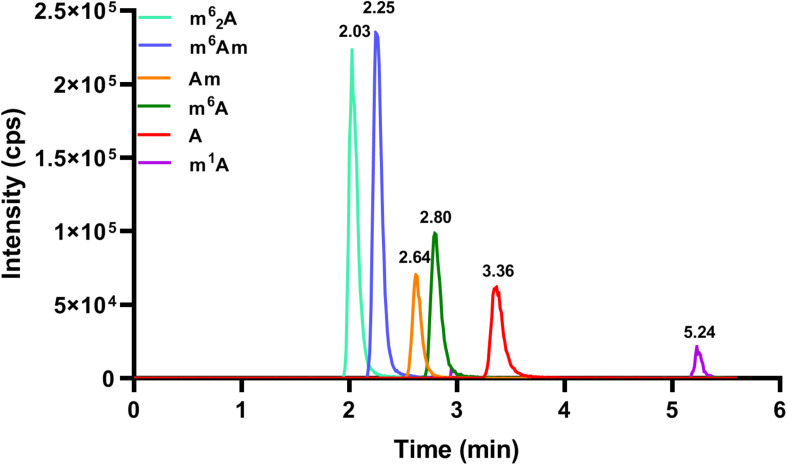
The MRM chromatograms of A, m^6^A, m^1^A, m^6^Am, Am, and m^6^_2_A standards. The concentration of each nucleoside standard was 25 nM, and the injection volume was 5.0 μl.

In order to achieve excellent chromatographic separation and obtain good chromatographic peak shape with appropriate retention, chromatographic conditions including the type of column and the composition of mobile phase were optimized. The chemical structures of adenosine and its methylated modifications are illustrated in Figure 1. Since m^1^A is positively charged, the retention of m^1^A on a C18 column is very weak. It is eluted from the C18 column quickly, and a symmetric peak shape is hard to obtain. As a complementary tool, HILIC is widely used for the separation of compounds with high polarity, and we found that m^1^A can be retained well on the HILIC column. Besides, a high proportion of organic solvent was used for HILIC separation, which could enhance the ionization efficiency of targets, leading to the improvement of detection sensitivity. Therefore, the BEH HILIC column (2.1 × 100 mm, 1.7 μm) was selected for subsequent analysis. In our previous study, we found that malic acid could enhance the detection of methylated nucleosides in HILIC–MS/MS (Guo et al., 2018a). Hence, malic acid was added into the mobile phase for the analysis of methylated adenosine modifications. As illustrated in Figure 2, the analysis can be finished within 6 min, and target nucleosides were perfectly separated, which suggested that the analytical method is quick, has high throughput, and is fit for large clinical samples.

To optimize the MRM parameters, a peristaltic pump was used, and the standard solution was directly infused into the mass spectrometer. For A, [M + H]^+^ ion at *m*/*z* 268.1 was observed in full-scan ESI-MS. Abundant [M + H]^+^ ions at *m*/*z* 282.1 were both observed for m^6^A and Am; for m^6^Am and m^6^_2_A, [M + H]^+^ ions at *m/z* 296.1 were both observed; and for m^1^A, M^+^ ion at *m*/*z* 282.1 was observed. Then, we carried out collision-induced dissociation (CID) experiments. According to the results of CID, for m^6^A, m^1^A, and m^6^Am, the most abundant fragment ions were all at *m*/*z* 150.0. For A and Am, the most abundant fragment ions were both at *m*/*z* 136.0, and for m^6^_2_A, the most abundant fragment ion was at *m*/*z* 164.0. From this point of view, the ion transitions *m*/*z* 268.1→136.0 (A), *m*/*z* 282.1→150.0 (m^6^A and m^1^A), *m*/*z* 282.1→136.0 (Am), *m*/*z* 296.1→150.0 (m^6^Am), and *m*/*z* 296.1→164.0 (m^6^_2_A) were utilized for quantitative determination. Similarly, the ion transitions *m*/*z* 273.1→136.0 ([^13^C_5_]A), *m*/*z* 285.1→153.0 ([D_3_]m^6^A and [D_3_]m^1^A), and *m*/*z* 299.1→153.0 ([D_3_]m^6^Am) were monitored. Additionally, mass spectrometry parameters containing DP, EP, CE, CXP, ion source temperature (TEM), ion spray voltage, ion source gas 1 (GS1), ion source gas 2 (GS2), and curtain gas (CUR) were optimized to enhance the sensitivity and are summarized in Supplementary Table 1. Under these optimized conditions, the limit of detection (LOD) value can reach 0.0025 nM (A), 0.01 nM (m^6^A), 0.25 nM (m^1^A), and 0.01 nM (m^6^Am), which are lower than the LOD values reported before (Djukovic et al., 2010; Chen et al., 2011; Guo et al., 2019), indicating that the analytical method has admirable sensitivity.

### Validation of Analytical Method

For method validation, we investigated the linearity, recovery, LOD, LOQ, intra-day and inter-day precision, and matrix effects. As shown in Table 2, the calibration curve of each analyte showed excellent linearity (*R*^2^ > 0.999) within a wide analytical range. The data of LOD and the LOQ of the analytes are also illustrated in Table 2, which indicated that the sensitivity of the developed method was superb. As for the matrix effect, calibration curves were also constructed in serum extracts and in pure solvent. The slope ratio values for A, m^6^A, m^1^A, and m^6^Am were 92.5, 96.9, 98.5, and 93.3%, respectively, which implied that the matrix had no interferences in this study.

**TABLE 2:** Linearities of A, m^6^A, m^1^A, and m^6^Am in the HILIC-MS/MS analysis method.

Compound	Linear equation	*R*^2^ value	Linear range (nM)	LOD (nM)	LOQ (nM)
A	*y* = 0.0302x + 0.0235	0.9998	0.5–100	0.0025	0.01
m^6^A	*y* = 0.1205x + 0.0182	0.9999	0.1–100	0.01	0.1
m^1^A	*y* = 0.0062x + 0.0053	1	1–1,000	0.25	0.5
m^6^Am	*y* = 0.7408x − 0.0032	1	0.05–5	0.01	0.025

As shown in Supplementary Table 3, recoveries ranged from 98.9 to 109.4%, indicating that satisfactory recovery was obtained. As shown in Supplementary Table 4, the intra-day precision value was within 6.0%, and the inter-day precision value was within 8.8%. The accuracy of the intra-day and inter-day analysis was in range of 89.0 to 105.5%. These data demonstrated that satisfactory reproducibility and accuracy were determined.

Moreover, to check the system stability after numbers of injections, QC samples were analyzed every 20 serum samples, and the accuracy and retention time were monitored. The results showed that the equipment system had an outstanding stability during measurement. In a word, all these results mentioned above revealed that the developed HILIC-MS/MS method is quick, sensitive, accurate, reliable, and reproducible. And it can meet the requirement for the targeted quantitative analysis of these nucleosides in serum samples.

### Quantification of Methylated Adenosine Modifications in Human Serum

**FIGURE 3:**
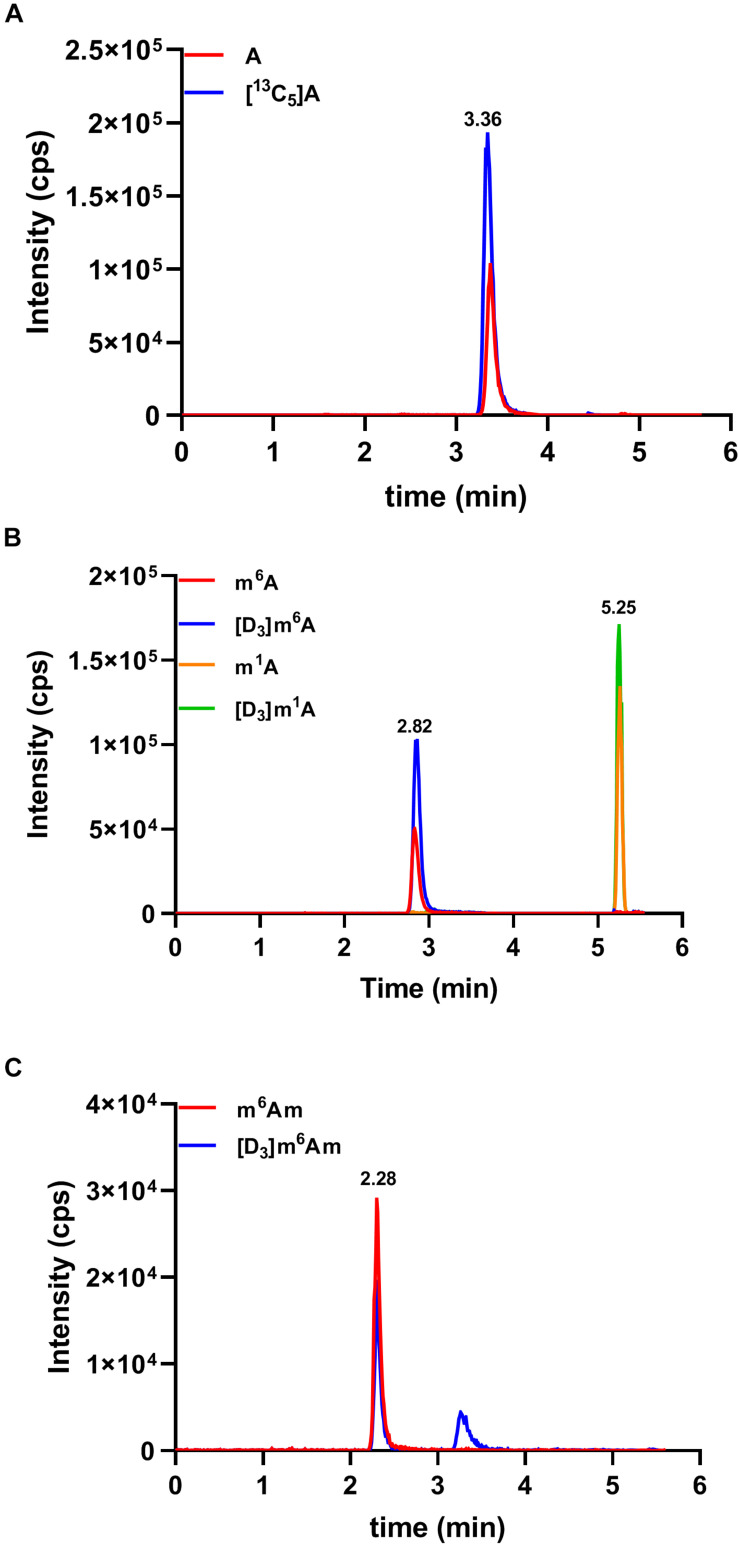
Representative MRM chromatograms of **(A)** A, **(B)** m^6^A and m^1^A, **(C)** m^6^Am, and spiked isotope-labeled internal standards in a serum sample.

By applying the developed HILIC-MS/MS method, we investigated the contents of these nucleosides in serum samples collected from 51 colorectal cancer patients, 27 gastric cancer patients, and 99 healthy controls. As demonstrated in Figure 3, the presence of A, m^6^A, m^1^A, and m^6^Am could be unquestionably confirmed in all serum samples since the retention time of these four nucleosides was identical to that of their corresponding internal standard, whereas Am and m^6^_2_A were not detected in all the serum samples due to their trace amounts in serum. The levels of A, m^6^A, m^1^A, and m^6^Am in serum samples were calculated according to the calibration curves, and the detailed concentrations were presented in Supplementary Table 2. In summary, the concentrations of A, m^6^A, m^1^A, and m^6^Am in human serum ranged from 1.95 to 34.19, 2.24 to 9.74, 115.16 to 215.77, and 0.16 to 2.56 nM, respectively.

### Content Change of Methylated Adenosine Modifications in Serum Samples From Cancer Patients

**FIGURE 4:**
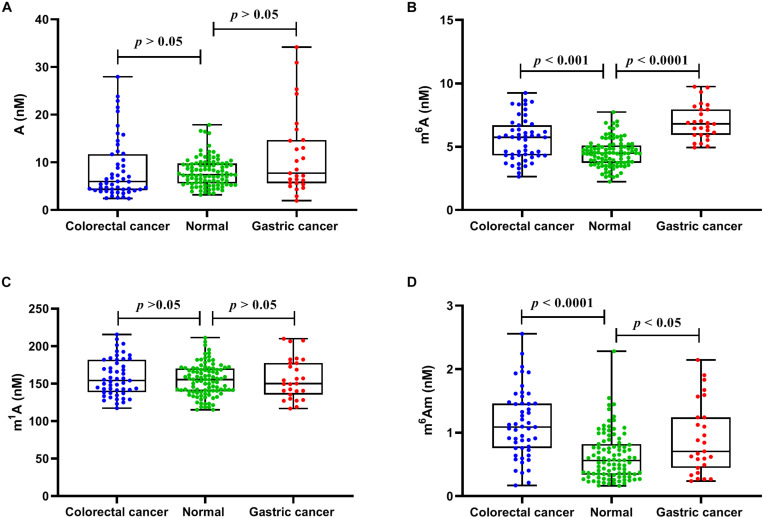
The measured concentrations of **(A)** A, **(B)** m^6^A, **(C)** m^1^A, and **(D)** m^6^Am in serum samples and statistical analysis.

We next evaluated whether there were differences in the concentrations of these nucleosides between healthy controls and cancer patients. In serum samples, the concentrations of A, m^6^A, m^1^A, and m^6^Am from healthy volunteers were in the range of 3.20–17.86, 2.24–7.73, 115.16–211.44, and 0.16–2.28 nM, respectively, and the average concentrations were 7.98 ± 3.01, 4.51 ± 1.08, 154.58 ± 21.10, and 0.63 ± 0.37 nM, respectively (*n* = 99). The concentrations of A, m^6^A, m^1^A, and m^6^Am in serum from patients with colorectal cancer were in the range of 2.41–27.94, 2.64–9.24, 117.45–215.77, and 0.17–2.56 nM, respectively, and the average concentrations were 8.47 ± 6.30, 5.57 ± 1.67, 158.62 ± 24.79, and 1.13 ± 0.53 nM, respectively (*n* = 51). For patients with gastric cancers, the concentrations of A, m^6^A, m^1^A, and m^6^Am in serum were in the range of 1.95–34.19, 4.94–9.74, 116.84–209.92, and 0.24–2.15 nM, respectively, and the average concentrations were 11.53 ± 8.55, 6.93 ± 1.38, 156.83 ± 27.07, and 0.91 ± 0.57 nM, respectively (*n* = 27). As illustrated in Figure 4, it is apparent that the levels of m^6^A and m^6^Am in serum were intensely increased in patients with colorectal cancer or gastric cancers, compared to those in healthy controls (colorectal cancer: *p* < 0.001 for m^6^A and *p* < 0.0001 for m^6^Am; gastric cancer: *p* < 0.0001 for m^6^A and *p* < 0.05 for m^6^Am). However, there was no difference in the levels of A and m^1^A between healthy controls and colorectal cancer or gastric cancer patients (*p* > 0.05).

**FIGURE 5:**
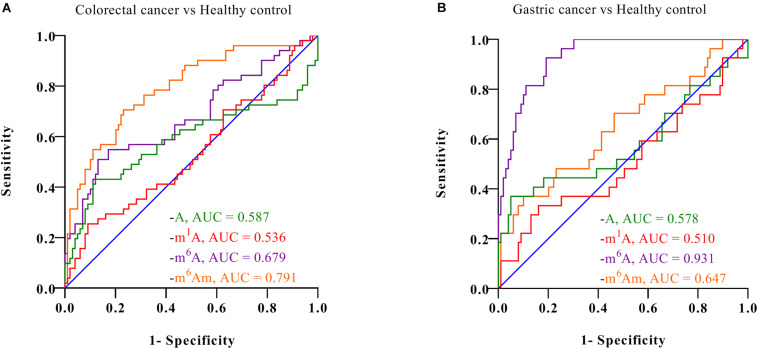
ROC analysis for **(A)** colorectal cancer vs. healthy controls and **(B)** gastric cancer vs. healthy controls.

Furthermore, ROC analysis was performed to evaluate the ability of these nucleosides to distinguish cancer patients from healthy controls. As demonstrated in Figure 5, for healthy volunteers and colorectal cancer patients, the area under the curve (AUC) for m^6^A and m^6^Am was 0.679 and 0.791, respectively. And for healthy controls and gastric cancer patients, the AUC was 0.931 and 0.647 for m^6^A and m^6^Am, respectively. These results indicated a correlation between the levels of m^6^A and m^6^Am in serum and the incidence of colorectal cancer and gastric cancer. Interestingly, we found that the AUC of m^6^Am was higher than that of m^6^A in colorectal cancer, whereas the AUC of m^6^A was higher than that of m^6^Am in gastric cancer, implying that m^6^Am and m^6^A were more effective indicators of colorectal cancer and gastric cancer, respectively. Colorectal cancer and gastric cancer are of a high incidence worldwide and has awfully high mortality, and early detection is extremely necessary. The results of this study revealed that the increase of m^6^A and m^6^Am in serum might have great potential to be novel non-invasive biomarkers for early detection of colorectal cancer and gastric cancer.

## Conclusion

In the present study, we developed a robust, sensitive, and trustworthy HILIC–MS/MS method for targeted quantitative analysis of A, m^6^A, m^1^A, Am, m^6^Am, and m^6^_2_A in human serum. By applying the established method, we successfully revealed, for the first time, the presence of m^6^Am in human serum and quantified the concentrations of A, m^6^A, m^1^A, and m^6^Am in 177 serum samples from three groups, namely, healthy volunteers, colorectal cancer patients, and gastric cancer patients. We found that the amount of m^6^A and m^6^Am were significantly higher in colorectal cancer or gastric cancer patients than those in healthy volunteers. Our data indicated that m^6^A and m^6^Am may serve as potential non-invasive biomarkers for early detection of colorectal cancer and gastric cancer. Furthermore, these results suggested that these methylated adenosine modifications might play important regulatory roles in the pathogenesis and progression of cancer.

## Data Availability Statement

The original contributions presented in the study are included in the article/Supplementary Material, further inquiries can be directed to the corresponding authors.

## Ethics Statement

The studies involving human participants were reviewed and approved by the Institutional Review Board of Medical Research, The Second Affiliated Hospital, Zhejiang University School of Medicine. The patients/participants provided their written informed consent to participate in this study.

## Author Contributions

CG and YY designed the study. YiH, CG, and ZF performed the experiments. ZF and JM collected the serum samples. YiH and CG analyzed and interpreted the data and wrote the manuscript. YaH and SZ edited the manuscript. All authors commented and approved the final manuscript.

## Conflict of Interest

The authors declare that the research was conducted in the absence of any commercial or financial relationships that could be construed as a potential conflict of interest.

## Publisher’s Note

All claims expressed in this article are solely those of the authors and do not necessarily represent those of their affiliated organizations, or those of the publisher, the editors and the reviewers. Any product that may be evaluated in this article, or claim that may be made by its manufacturer, is not guaranteed or endorsed by the publisher.
