# Supplementary material for: Quantitative Analysis of Methylated Adenosine Modifications Revealed Increased Levels of N6-Methyladenosine (m6A) and N6,2′-O-Dimethyladenosine (m6Am) in Serum From Colorectal Cancer and Gastric Cancer Patients
Source: Front Cell Dev Biol. 2021 Jul 26;9:694673. doi: 10.3389/fcell.2021.694673 (PMC8350345; doi:10.3389/fcell.2021.694673)
Supplement: Supplementary file 1 [file Data_Sheet_1.docx]

**Quantitative analysis of methylated adenosine modifications revealed increased levels of *N*^6^-methyladenosine (m^6^A) and *N*^6^,2′-O-dimethyladenosine (m^6^Am) in serum from colorectal cancer and gastric cancer patients**

Yiqiu Hu^1^, Zhihao Fang^1^, Jiayi Mu^1^, Yanqin Huang^1^, Shu Zheng^1^, Ying Yuan^2^*, Cheng Guo^1^*

^1^Cancer Institute (Key Laboratory of Cancer Prevention and Intervention, China National Ministry of Education), The Second Affiliated Hospital, Zhejiang University School of Medicine, Hangzhou, Zhejiang 310009, China

^2^Department of Medical Oncology, The Second Affiliated Hospital, Zhejiang University School of Medicine, Hangzhou, Zhejiang 310009, China

*Corresponding author:

Ying Yuan, yuanying1999@zju.edu.cn;

Cheng Guo, cheng_guo@zju.edu.cn

Tel.: +86-571-87784501; Fax: +86-571-87214404.

**Supplementary Information**

**Table S1.** The optimized MRM parameters for methylated adenosine modifications and isotope-labelled internal standards.

**Table S2.** Levels of A, m^6^A, m^1^A and m^6^Am in serum from healthy controls, colorectal cancer patients and gastric cancer patients.

**Table S3.** Recovery of the HILIC-MS/MS method obtained at three different spiking levels.

**Table S4.** Precision (intra- and interday) and accuracy of the developed HILIC-MS/MS method for the analysis of A, m^6^A, m^1^A and m^6^Am.

**Table S1.** The optimized MRM parameters for methylated adenosine modifications and isotope-labelled internal standards.

| **Compound** | **DP (V)** | **EP (V)** | **CE (V)** | **CXP (V)** |
| --- | --- | --- | --- | --- |
| A | 50 | 10 | 23 | 10 |
| [^13^C_5_]A | 50 | 10 | 21 | 10 |
| m^6^A | 55 | 10 | 25 | 10 |
| [D_3_]m^6^A | 50 | 10 | 27 | 10 |
| m^1^A | 45 | 8 | 23 | 10 |
| [D_3_]m^1^A | 50 | 10 | 27 | 10 |
| Am | 60 | 10 | 24 | 6 |
| m^6^Am | 50 | 10 | 25 | 10 |
| [D_3_]m^6^Am | 50 | 10 | 25 | 10 |
| m^6^_2_A | 55 | 10 | 28 | 8 |

*DP* declustering potential, *CE* collision energy, *EP* entrance potential, *CXP* collision cell exit potential

**Table S2.** Levels of A, m^6^A, m^1^A and m^6^Am in serum from healthy controls, colorectal cancer patients and gastric cancer patients.

| **NO.** | **A**  **(nmol/l )** | **m^6^A**  **(nmol/l)** | **m^1^A**  **(nmol/l)** | **m^6^Am**  **(nmol/l)** |
| --- | --- | --- | --- | --- |
| N1 | 6.99 ± 0.57 | 3.43 ± 0.05 | 172.28 ± 0.09 | 1.25 ± 0.09 |
| N2 | 5.59 ± 0.03 | 5.13 ± 0.15 | 153.41 ± 1.69 | 0.71 ± 0.02 |
| N3 | 5.37 ± 0.05 | 4.98 ± 0.06 | 142.41 ± 2.81 | 1.45 ± 0.01 |
| N4 | 4.87 ± 0.06 | 4.65 ± 0.05 | 141.34 ± 2.49 | 0.99 ± 0.08 |
| N5 | 4.41 ± 0.14 | 3.92 ± 0.07 | 132.50 ± 1.89 | 0.82 ± 0.00 |
| N6 | 4.02 ± 0.19 | 4.46 ± 0.07 | 178.36 ± 1.50 | 0.31 ± 0.00 |
| N7 | 5.74 ± 0.06 | 5.23 ± 0.04 | 174.87 ± 4.28 | 0.47 ± 0.03 |
| N8 | 8.67 ± 0.06 | 4.42 ± 0.16 | 167.89 ± 1.39 | 0.60 ± 0.01 |
| N9 | 9.86 ± 0.20 | 3.55 ± 0.01 | 150.41 ± 7.52 | 0.17 ± 0.00 |
| N10 | 9.65 ± 0.10 | 4.44 ± 0.04 | 141.37 ± 4.76 | 0.27 ± 0.00 |
| N11 | 13.49 ± 0.09 | 5.77 ± 0.07 | 195.39 ± 3.34 | 0.20 ± 0.01 |
| N12 | 7.63 ± 0.24 | 3.93 ± 0.02 | 139.26 ± 1.08 | 0.20 ± 0.00 |
| N13 | 9.36 ± 0.18 | 4.78 ± 0.06 | 156.56 ± 2.01 | 0.69 ± 0.03 |
| N14 | 7.37 ± 0.21 | 5.67 ± 0.17 | 167.73 ± 9.14 | 0.44 ± 0.01 |
| N15 | 8.94 ± 0.37 | 4.24 ± 0.19 | 136.58 ± 5.90 | 0.26 ± 0.01 |
| N16 | 16.45 ± 0.04 | 5.83 ± 0.03 | 182.22 ± 0.11 | 0.49 ± 0.02 |
| N17 | 8.25 ± 0.05 | 5.72 ± 0.28 | 159.08 ± 2.99 | 0.25 ± 0.00 |
| N18 | 11.15 ± 0.33 | 5.67 ± 0.06 | 140.72 ± 1.89 | 0.20 ± 0.00 |
| N19 | 4.46 ± 0.03 | 4.13 ± 0.03 | 118.85 ± 4.40 | 0.26 ± 0.00 |
| N20 | 11.12 ± 0.30 | 5.00 ± 0.07 | 159.43 ± 1.55 | 0.75 ± 0.03 |
| N21 | 8.38 ± 0.02 | 4.12 ± 0.07 | 165.48 ± 2.07 | 0.24 ± 0.01 |
| N22 | 11.47 ± 0.13 | 4.05 ± 0.14 | 155.27 ± 6.05 | 0.26 ± 0.02 |
| N23 | 10.02 ± 0.17 | 5.18 ± 0.23 | 155.78 ± 1.51 | 0.30 ± 0.00 |
| N24 | 17.86 ± 0.25 | 4.51 ± 0.03 | 141.52 ± 3.19 | 0.77 ± 0.04 |
| N25 | 9.43 ± 0.22 | 3.81 ± 0.03 | 133.41 ± 20.57 | 0.37 ± 0.01 |
| N26 | 11.61 ± 0.12 | 4.98 ± 0.18 | 190.89 ± 4.11 | 0.23 ± 0.02 |
| N27 | 10.76 ± 0.12 | 5.23 ± 0.08 | 157.99 ± 6.37 | 0.48 ± 0.00 |
| N28 | 6.70 ± 0.21 | 4.86 ± 0.04 | 118.62 ± 17.39 | 1.06 ± 0.02 |
| N29 | 5.53 ± 0.10 | 4.64 ± 0.09 | 176.22 ± 7.77 | 0.81 ± 0.00 |
| N30 | 9.76 ± 0.08 | 6.85 ± 0.00 | 205.91 ± 7.42 | 0.33 ± 0.01 |
| N31 | 7.42 ± 0.31 | 4.52 ± 0.02 | 140.46 ± 5.01 | 0.27 ± 0.00 |
| N32 | 12.27 ± 0.19 | 6.67 ± 0.01 | 170.98 ± 4.92 | 1.00 ± 0.03 |
| N33 | 12.21 ± 0.19 | 4.85 ± 0.03 | 211.44 ± 5.83 | 0.76 ± 0.01 |
| N34 | 8.48 ± 0.013 | 4.52 ± 0.04 | 184.28 ± 10.13 | 1.20 ± 0.03 |
| N35 | 9.79 ± 0.07 | 4.54 ± 0.07 | 163.80 ± 6.10 | 0.69 ± 0.02 |
| N36 | 5.96 ± 0.01 | 3.76 ± 0.13 | 125.17 ± 13.02 | 0.96 ± 0.02 |
| N37 | 16.12 ± 0.21 | 3.23 ± 0.07 | 136.67 ± 14.40 | 0.61 ± 0.06 |
| N38 | 8.34 ± 0.12 | 3.00 ± 0.02 | 131.79 ± 10.25 | 1.10 ± 0.00 |
| N39 | 10.66 ± 0.06 | 3.70 ± 0.02 | 132.10 ± 8.34 | 1.08 ± 0.08 |
| N40 | 10.10 ± 0.32 | 4.46 ± 0.12 | 170.65 ± 10.47 | 0.99 ± 0.01 |
| N41 | 12.53 ± 0.56 | 4.40 ± 0.03 | 152.64 ± 7.13 | 0.87 ± 0.01 |
| N42 | 16.63 ± 0.15 | 3.43 ± 0.01 | 135.14 ± 5.69 | 1.44 ± 0.02 |
| N43 | 10.11 ± 0.14 | 4.68 ± 0.06 | 166.45 ± 17.95 | 0.35 ± 0.01 |
| N44 | 6.54 ± 0.15 | 3.94 ± 0.00 | 144.87 ± 26.02 | 0.73 ± 0.00 |
| N45 | 9.32 ± 0.11 | 4.05 ± 0.02 | 123.11 ± 8.46 | 0.84 ± 0.11 |
| N46 | 8.22 ± 0.08 | 2.69 ± 0.04 | 155.63 ± 9.31 | 1.19 ± 0.05 |
| N47 | 8.01 ± 0.00 | 4.49 ± 0.02 | 171.81 ± 10.97 | 0.53 ± 0.04 |
| N48 | 10.40 ± 0.43 | 3.38 ± 0.15 | 190.01 ± 8.10 | 0.66 ± 0.02 |
| N49 | 10.46 ± 0.35 | 4.48 ± 0.02 | 166.13 ± 6.91 | 0.64 ± 0.01 |
| N50 | 8.53 ± 0.05 | 2.82 ± 0.04 | 153.51 ± 8.47 | 0.73 ± 0.02 |
| N51 | 10.03 ± 0.10 | 2.95 ± 0.01 | 115.27 ± 6.18 | 0.37 ± 0.00 |
| N52 | 6.48 ± 0.27 | 4.53 ± 0.04 | 165.84 ± 9.64 | 0.78 ± 0.04 |
| N53 | 7.77 ± 0.09 | 3.70 ± 0.01 | 142.54 ± 5.52 | 0.65 ± 0.00 |
| N54 | 4.73 ± 0.21 | 2.24 ± 0.08 | 121.56 ± 15.83 | 0.89 ± 0.05 |
| N55 | 6.99 ± 0.33 | 4.04 ± 0.42 | 145.40 ± 7.21 | 1.04 ± 0.03 |
| N56 | 11.07 ± 0.10 | 3.72 ± 0.14 | 130.86 ± 5.64 | 0.29 ± 0.00 |
| N57 | 9.81 ± 0.15 | 3.74 ± 0.34 | 137.81 ± 8.63 | 0.35 ± 0.01 |
| N58 | 5.22 ± 0.07 | 3.48 ± 0.13 | 135.17 ± 8.35 | 0.50 ± 0.05 |
| N59 | 5.65 ± 0.25 | 3.26 ± 0.17 | 128.96 ± 9.41 | 0.78 ± 0.01 |
| N60 | 6.16 ± 0.04 | 3.50 ± 0.21 | 180.98 ± 18.87 | 1.06 ± 0.07 |
| N61 | 4.12 ± 0.11 | 2.60 ± 0.08 | 133.79 ± 2.57 | 0.30 ± 0.02 |
| N62 | 5.24 ± 0.01 | 3.57 ± 0.07 | 160.83 ± 11.86 | 0.16 ± 0.01 |
| N63 | 7.44 ± 0.02 | 2.61 ± 0.04 | 142.39 ± 5.57 | 0.30 ± 0.02 |
| N64 | 8.51 ± 0.25 | 6.89 ± 0.24 | 174.71 ± 3.14 | 0.60 ± 0.01 |
| N65 | 7.16 ± 0.02 | 2.91 ± 0.06 | 172.59 ± 2.80 | 0.26 ± 0.01 |
| N66 | 5.78 ± 0.00 | 6.44 ± 0.18 | 181.66 ± 0.55 | 0.50 ± 0.03 |
| N67 | 6.13 ± 0.09 | 4.46 ± 0.02 | 145.82 ± 15.50 | 0.36 ± 0.01 |
| N68 | 9.15 ± 0.22 | 5.54 ± 0.05 | 170.93 ± 11.14 | 0.57 ± 0.00 |
| N69 | 5.22 ± 0.17 | 4.77 ± 0.09 | 149.91 ± 8.84 | 0.73 ± 0.03 |
| N70 | 6.38 ± 0.03 | 5.94 ± 0.13 | 170.38 ± 4.03 | 0.28 ± 0.02 |
| N71 | 9.25 ± 0.49 | 4.32 ± 0.01 | 160.32 ± 19.42 | 0.21 ± 0.01 |
| N72 | 4.46 ± 0.08 | 5.19 ± 0.00 | 139.98 ± 7.67 | 0.40 ± 0.01 |
| N73 | 5.14 ± 0.15 | 3.44 ± 0.01 | 168.62 ± 14.86 | 0.36 ± 0.02 |
| N74 | 3.46 ± 0.14 | 3.79 ± 0.03 | 186.21 ± 1.68 | 1.55 ± 0.01 |
| N75 | 4.81 ± 0.20 | 3.59 ± 0.03 | 151.91 ± 7.40 | 0.61 ± 0.04 |
| N76 | 7.18 ± 0.45 | 5.71 ± 0.01 | 168.67 ± 16.86 | 0.43 ± 0.01 |
| N77 | 4.85 ± 0.20 | 4.97 ± 0.01 | 159.07 ± 9.61 | 0.44 ± 0.01 |
| N78 | 5.09 ± 0.07 | 2.71 ± 0.09 | 156.99 ± 10.47 | 0.58 ± 0.02 |
| N79 | 3.73 ± 0.29 | 3.54 ± 0..13 | 168.63 ± 12.53 | 0.53 ± 0.00 |
| N80 | 7.03 ± 0.02 | 5.10 ± 0.02 | 146.54 ± 10.42 | 0.23 ± 0.01 |
| N81 | 11.41 ± 0.18 | 4.83 ± 0.01 | 139.86 ± 3.74 | 0.53 ± 0.02 |
| N82 | 7.39 ± 0.20 | 7.73 ± 0.03 | 201.36 ± 21.73 | 2.28 ± 0.01 |
| N83 | 6.99 ± 0.16 | 7.00 ± 0.02 | 181.82 ± 21.17 | 1.37 ± 0.04 |
| N84 | 9.21 ± 0.06 | 5.27 ± 0.04 | 142.30 ± 12.05 | 0.43 ± 0.00 |
| N85 | 9.56 ± 0.09 | 5.89 ± 0.04 | 158.94 ± 19.48 | 0.23 ± 0.01 |
| N86 | 9.40 ± 0.24 | 4.91 ± 0.20 | 123.22 ± 4.23 | 0.56 ± 0.02 |
| N87 | 6.47 ± 0.22 | 4.94 ± 0.13 | 141.96 ± 19.84 | 0.57 ± 0.01 |
| N88 | 5.35 ± 0.02 | 4.26 ± 0.04 | 161.65 ± 11.03 | 0.35 ± 0.00 |
| N89 | 3.89 ± 0.23 | 5.98 ± 0.06 | 171.62 ± 8.22 | 0.60 ± 0.01 |
| N90 | 7.34 ± 0.36 | 6.51 ± 0.01 | 164.00 ± 11.25 | 0.91 ± 0.03 |
| N91 | 7.31 ± 0.19 | 4.85 ± 0.03 | 147.21 ± 2.73 | 0.41 ± 0.00 |
| N92 | 4.25 ± 0.01 | 4.84 ± 0.07 | 141.92 ± 0.93 | 1.06 ± 0.02 |
| N93 | 6.15 ± 0.11 | 4.75 ± 0.14 | 119.21 ± 3.11 | 1.11 ± 0.05 |
| N94 | 7.10 ± 0.07 | 4.50 ± 0.04 | 115.16 ± 4.81 | 0.98 ± 0.02 |
| N95 | 3.20 ± 0.21 | 4.70 ± 0.01 | 123.63 ± 3.06 | 0.35 ± 0.02 |
| N96 | 5.28 ± 0.19 | 3.49 ± 0.06 | 156.99 ± 17.10 | 0.33 ± 0.02 |
| N97 | 8.97 ± 0.20 | 6.02 ± 0.09 | 139.10 ± 5.54 | 0.37 ± 0.00 |
| N98 | 3.52 ± 0.01 | 3.82 ± 0.10 | 170.91 ± 5.59 | 0.38 ± 0.00 |
| N99 | 6.02 ± 0.29 | 3.94 ± 0.13 | 149.76 ± 7.64 | 0.78 ± 0.01 |
| C1 | 20.74 ± 0.04 | 4.11 ± 0.01 | 156.69 ± 17.60 | 1.31 ± 0.01 |
| C2 | 13.71 ± 0.07 | 5.95 ± 0.05 | 131.63 ± 7.44 | 1.96 ± 0.03 |
| C3 | 3.49 ± 0.12 | 4.61 ± 0.13 | 117.45 ± 11.92 | 0.91 ± 0.04 |
| C4 | 9.26 ± 0.06 | 5.55 ± 0.11 | 155.42 ± 8.83 | 1.45 ± 0.02 |
| C5 | 8.83 ± 0.15 | 6.18 ± 0.22 | 144.90 ± 7.09 | 1.74 ± 0.04 |
| C6 | 4.70 ± 0.05 | 4.34 ± 0.02 | 177.30 ± 1.07 | 0.76 ± 0.05 |
| C7 | 4.15 ± 0.14 | 4.72 ± 0.08 | 168.49 ± 2.04 | 1.46 ± 0.03 |
| C8 | 10.49 ± 0.08 | 5.05 ± 0.08 | 123.10 ± 8.01 | 0.78 ± 0.01 |
| C9 | 5.97 ± 0.11 | 8.34 ± 0.02 | 183.17 ± 15.35 | 1.46 ± 0.00 |
| C10 | 6.74 ± 0.03 | 4.39 ± 0.18 | 157.19 ± 6.58 | 0.95 ± 0.03 |
| C11 | 4.71 ± 0.03 | 8.40 ± 0.28 | 203.34 ± 5.07 | 1.45 ± 0.03 |
| C12 | 11.70 ± 0.10 | 3.69 ± 0.03 | 137.68 ± 0.60 | 0.17 ± 0.00 |
| C13 | 4.46 ± 0.11 | 8.55 ± 0.09 | 209.35 ± 12.01 | 2.56 ± 0.06 |
| C14 | 6.02 ± 0.13 | 5.90 ± 0.07 | 141.96 ± 2.42 | 1.07 ± 0.01 |
| C15 | 9.58 ± 0.42 | 7.57 ± 0.05 | 182.10 ± 5.24 | 1.57 ± 0.03 |
| C16 | 3.68 ± 0.07 | 9.24 ± 0.21 | 201.59 ± 10.27 | 1.33 ± 0.05 |
| C17 | 3.70 ± 0.07 | 5.63 ± 0.08 | 149.99 ± 6.46 | 1.04 ± 0.00 |
| C18 | 5.41 ± 0.09 | 6.15 ± 0.05 | 192.54 ± 6.02 | 2.04 ± 0.04 |
| C19 | 15.81 ± 0.08 | 2.64 ± 0.03 | 143.01 ± 5.62 | 0.73 ± 0.01 |
| C20 | 2.70 ± 0.02 | 8.35 ± 0.17 | 188.14 ± 4.52 | 1.93 ± 0.07 |
| C21 | 4.33 ± 0.04 | 5.75 ± 0.13 | 173.08 ± 21.17 | 0.89 ± 0.04 |
| C22 | 3.79 ± 0.08 | 4.30 ± 0.00 | 184.18 ± 4.30 | 0.88 ± 0.03 |
| C23 | 3.97 ± 0.09 | 6.36 ± 0.15 | 137.31 ± 3.15 | 0.85 ± 0.06 |
| C24 | 7.27 ± 0.04 | 4.31 ± 0.01 | 165.85 ± 1.53 | 0.37 ± 0.00 |
| C25 | 2.59 ± 0.01 | 3.29 ± 0.01 | 170.77 ± 4.93 | 1.09 ± 0.02 |
| C26 | 7.03 ± 0.03 | 3.46 ± 0.01 | 183.42 ± 1.33 | 0.40 ± 0.01 |
| C27 | 2.94 ± 0.03 | 5.89 ± 0.01 | 133.59 ± 4.96 | 1.62 ± 0.05 |
| C28 | 2.41 ± 0.00 | 4.51 ± 0.05 | 193.24 ± 25.27 | 2.25 ± 0.05 |
| C29 | 4.11 ± 0.13 | 4.61 ± 0.08 | 151.12 ± 3.02 | 1.10 ± 0.06 |
| C30 | 4.50 ± 0.19 | 4.16 ± 0.04 | 143.64 ± 5.26 | 0.40 ± 0.01 |
| C31 | 2.49 ± 0.14 | 2.92 ± 0.04 | 138.56 ± 6.59 | 1.21 ± 0.02 |
| C32 | 4.73 ± 0.22 | 7.50 ± 0.12 | 182.00 ± 2.20 | 0.58 ± 0.08 |
| C33 | 27.94 ± 1.20 | 8.66 ± 0.13 | 215.77 ± 2.01 | 0.64 ± 0.03 |
| C34 | 14.54 ± 0.10 | 6.70 ± 0.07 | 148.68 ± 3.03 | 0.53 ± 0.01 |
| C35 | 6.52 ± 0.03 | 4.40 ± 0.10 | 187.96 ± 12.84 | 1.95 ± 0.02 |
| C36 | 22.91 ± 0.29 | 3.68 ± 0.05 | 152.85 ± 4.10 | 1.07 ± 0.04 |
| C37 | 6.49 ± 0.09 | 4.61 ± 0.03 | 163.36 ± 11.43 | 0.64 ± 0.01 |
| C38 | 3.89 ± 0.15 | 3.89 ± 0.10 | 158.04 ± 0.29 | 0.91 ± 0.07 |
| C39 | 16.07 ± 0.01 | 6.79 ± 0.02 | 169.65 ± 4.28 | 0.21 ± 0.00 |
| C40 | 12.33 ± 0.10 | 7.01 ± 0.26 | 166.04 ± 2.65 | 1.27 ± 0.05 |
| C41 | 21.56 ± 0.20 | 6.31 ± 0.08 | 141.41 ± 4.72 | 1.37 ± 0.01 |
| C42 | 4.39 ± 0.21 | 3.49 ± 0.03 | 142.90 ± 1.61 | 1.15 ± 0.01 |
| C43 | 4.75 ± 0.03 | 3.63 ± 0.06 | 154.28 ± 7.46 | 0.76 ± 0.01 |
| C44 | 8.11 ± 0.15 | 4.33 ± 0.03 | 134.57 ± 3.53 | 0.91 ± 0.01 |
| C45 | 5.36 ± 0.03 | 6.08 ± 0.03 | 129.08 ± 12.61 | 1.62 ± 0.13 |
| C46 | 5.65 ± 0.03 | 5.82 ± 0.00 | 127.63 ± 6.26 | 0.60 ± 0.02 |
| C47 | 4.34 ± 0.00 | 5.77 ± 0.09 | 129.23 ± 3.42 | 1.14 ± 0.00 |
| C48 | 11.83 ± 0.14 | 6.90 ± 0.12 | 148.03 ± 2.73 | 1.64 ± 0.00 |
| C49 | 17.70 ± 0.44 | 7.94 ± 4.68 | 143.44 ± 10.74 | 1.13 ± 0.04 |
| C50 | 7.50 ± 0.10 | 5.87 ± 0.13 | 125.13 ± 14.17 | 0.57 ± 0.04 |
| C51 | 23.85 ± 0.39 | 5.74 ± 0.13 | 129.97 ± 3.24 | 1.48 ± 0.07 |
| G1 | 14.69 ± 0.12 | 8.19 ± 0.06 | 177.69 ± 2.52 | 0.63 ± 0.02 |
| G2 | 12.77 ± 0.74 | 5.24 ± 0.25 | 127.30 ± 1.64 | 1.57 ± 0.00 |
| G3 | 25.36 ± 1.92 | 9.74 ± 0.84 | 209.92 ± 3.45 | 0.27 ± 0.01 |
| G4 | 18.17 ± 0.01 | 6.29 ± 0.02 | 128.24 ± 15.58 | 0.24 ± 0.00 |
| G5 | 5.71 ± 0.01 | 7.94 ± 0.19 | 183.59 ± 10.54 | 2.15 ± 0.02 |
| G6 | 2.91 ± 0.13 | 5.53 ± 0.00 | 127.38 ± 6.19 | 0.62 ± 0.01 |
| G7 | 5.39 ± 0.03 | 8.42 ± 0.04 | 182.17 ± 22.12 | 0.58 ± 0.00 |
| G8 | 13.06 ± 0.34 | 6.87 ± 0.05 | 149.39 ± 18.42 | 0.96 ± 0.01 |
| G9 | 24.38 ± 0.48 | 8.30 ± 0.15 | 177.31 ± 0.18 | 1.24 ± 0.03 |
| G10 | 7.14 ± 0.03 | 6.96 ± 0.27 | 156.55 ± 0.35 | 1.67 ± 0.01 |
| G11 | 10.89 ± 0.08 | 5.24 ± 0.08 | 140.74 ± 9.65 | 0.27 ± 0.02 |
| G12 | 10.29 ± 0.10 | 6.47 ± 0.12 | 130.01 ± 4.65 | 1.91 ± 0.05 |
| G13 | 4.99 ± 0.05 | 4.94 ± 0.04 | 116.84 ± 0.98 | 0.67 ± 0.02 |
| G14 | 1.95 ± 0.09 | 6.44 ± 0.01 | 154.60 ± 15.51 | 1.24 ± 0.01 |
| G15 | 4.64 ± 0.00 | 6.89 ± 0.10 | 142.51 ± 18.84 | 0.45 ± 0.01 |
| G16 | 16.94 ± 0.17 | 6.79 ± 0.11 | 168.80 ± 6.98 | 0.33 ± 0.01 |
| G17 | 6.46 ± 0.01 | 6.57 ± 0.12 | 149.58 ± 16.12 | 1.12 ± 0.03 |
| G18 | 6.22 ± 0.13 | 9.32 ± 0.04 | 207.61 ± 11.43 | 1.59 ± 0.03 |
| G19 | 7.73 ± 0.29 | 6.12 ± 0.09 | 135.43 ± 1.13 | 0.59 ± 0.01 |
| G20 | 7.21 ± 0.04 | 7.93 ± 0.11 | 182.33 ± 20.04 | 1.83 ± 0.02 |
| G21 | 14.55 ± 0.22 | 5.94 ± 0.12 | 140.05 ± 0.74 | 0.49 ± 0.03 |
| G22 | 6.46 ± 0.23 | 5.86 ± 0.00 | 157.18 ± 7.40 | 1.10 ± 0.03 |
| G23 | 4.35 ± 0.14 | 7.57 ± 0.02 | 150.22 ± 18.04 | 0.88 ± 0.03 |
| G24 | 8.51 ± 0.26 | 7.02 ± 0.04 | 140.73 ± 14.22 | 0.84 ± 0.05 |
| G25 | 34.19 ± 0.61 | 9.68 ± 0.05 | 206.59 ± 2.89 | 0.71 ± 0.00 |
| G26 | 5.62 ± 0.06 | 5.04 ± 0.05 | 119.07 ± 0.87 | 0.27 ± 0.01 |
| G27 | 30.93 ± 0.08 | 5.92 ± 0.04 | 172.79 ± 15.78 | 0.37 ± 0.01 |

**Table S3.** Recovery of the HILIC-MS/MS method obtained at three different spiking levels.

|  | **Spiked amount (nM)** | **Mean ± SD**  **(nM)** | **Average recovery (%)** | **RSD**  **(%)** |
| --- | --- | --- | --- | --- |
| A | 0 | 2.02 ± 0.01 | - | 0.35 |
|  | 1 (Low) | 3.02 ± 0.02 | 100.87 | 0.82 |
|  | 5 (Medium) | 7.04 ± 0.16 | 100.57 | 2.21 |
|  | 50 (High) | 52.80 ± 0.44 | 101.56 | 0.83 |
|  |  |  |  |  |
| m^6^A | 0 | 12.07 ± 0.16 | - | 1.35 |
|  | 1 (Low) | 13.06 ± 0.06 | 98.86 | 0.49 |
|  | 5 (Medium) | 17.29 ± 0.19 | 103.84 | 1.10 |
|  | 50 (High) | 66.75 ± 0.11 | 109.35 | 0.17 |
|  |  |  |  |  |
| m^1^A | 0 | 50.94 ± 2.90 | - | 5.70 |
|  | 10 (Low) | 61.18 ± 3.18 | 102.48 | 5.20 |
|  | 100 (Medium) | 157.89 ± 7.63 | 106.95 | 4.83 |
|  | 500 (High) | 563.25 ± 3.83 | 102.46 | 0.68 |
|  |  |  |  |  |
| m^6^Am | 0 | 0.35 ± 0.00 | - | 0.24 |
|  | 0.1 (Low) | 0.45 ± 0.02 | 101.12 | 3.57 |
|  | 1 (Medium) | 1.34 ± 0.00 | 99.49 | 0.10 |
|  | 5 (High) | 5.36 ± 0.06 | 100.34 | 1.09 |

**Table S4.** Precision (intra- and interday) and accuracy of the developed HILIC-MS/MS method for the analysis of A, m^6^A, m^1^A and m^6^Am.

| **QC** | **Theoretical values (nM)** | **Intra-day (n = 9)** | | | | **Inter-day (n = 3)** | | |
| --- | --- | --- | --- | --- | --- | --- | --- | --- |
|  |  | Mean ± SD (nM) | | RSD (%) | Accuracy (%) | Mean ± SD (nM) | RSD (%) | Accuracy  (%) |
| A | 1 (Low) | 1.01 ± 0.07 | | 6.70 | 100.53 | 0.98 ± 0.06 | 6.06 | 97.74 |
|  | 10 (Medium) | 9.75 ± 0.13 | | 1.33 | 97.50 | 9.76 ± 0.18 | 1.82 | 97.60 |
|  | 75 (High) | 74.95 ± 1.31 | | 1.75 | 99.93 | 74.83 ± 1.31 | 1.75 | 99.80 |
|  |  |  |  | |  |  |  |  |
| m^6^A | 1 (Low) | 0.90 ± 0.03 | | 2.86 | 90.37 | 0.90 ± 0.03 | 3.77 | 88.95 |
|  | 10 (Medium) | 10.38 ± 0.13 | | 1.30 | 103.77 | 10.38 ± 0.15 | 1.46 | 103.79 |
|  | 75 (High) | 76.27 ± 2.04 | | 2.68 | 101.70 | 76.54 ± 2.07 | 2.71 | 102.05 |
|  |  |  | |  |  |  |  |  |
| m^1^A | 10 (Low) | 9.87 ± 0.99 | | 6.18 | 95.36 | 9.47 ± 0.81 | 8.56 | 94.73 |
|  | 250 (Medium) | 246.52 ± 11.87 | | 2.83 | 98.61 | 246.06 ± 10.30 | 4.23 | 98.41 |
|  | 750 (High) | 775.96 ± 32.12 | | 4.14 | 103.46 | 775.68 ± 34.03 | 4.39 | 103.42 |
|  |  |  | |  |  |  |  |  |
| m^6^Am | 0.1 (Low) | 0.10 ± 0.01 | | 6.05 | 103.15 | 0.10 ± 0.01 | 8.82 | 102.37 |
|  | 2.5 (Medium) | 2.63 ± 0.05 | | 1.74 | 105.36 | 2.64 ± 0.06 | 2.17 | 105.48 |
|  | 25 (High) | 24.10 ± 0.48 | | 2.01 | 96.39 | 24.12 ± 0.53 | 2.18 | 96.49 |
